# Stroke-induced damage on the blood–brain barrier

**DOI:** 10.3389/fneur.2023.1248970

**Published:** 2023-09-28

**Authors:** Song Xue, Xin Zhou, Zhi-Hui Yang, Xiang-Kun Si, Xin Sun

**Affiliations:** Stroke Center, Department of Neurology, The First Hospital of Jilin University, Changchun, China

**Keywords:** blood–brain barrier, neurovascular unit, microvascular endothelial cells, astrocytes, ischemic stroke

## Abstract

The blood–brain barrier (BBB) is a functional phenotype exhibited by the neurovascular unit (NVU). It is maintained and regulated by the interaction between cellular and non-cellular matrix components of the NVU. The BBB plays a vital role in maintaining the dynamic stability of the intracerebral microenvironment as a barrier layer at the critical interface between the blood and neural tissues. The large contact area (approximately 20 m^2^/1.3 kg brain) and short diffusion distance between neurons and capillaries allow endothelial cells to dominate the regulatory role. The NVU is a structural component of the BBB. Individual cells and components of the NVU work together to maintain BBB stability. One of the hallmarks of acute ischemic stroke is the disruption of the BBB, including impaired function of the tight junction and other molecules, as well as increased BBB permeability, leading to brain edema and a range of clinical symptoms. This review summarizes the cellular composition of the BBB and describes the protein composition of the barrier functional junction complex and the mechanisms regulating acute ischemic stroke-induced BBB disruption.

## 1. Introduction

Proper central nervous system (CNS) functioning requires a highly specific and dynamically stable intracerebral microenvironment with extremely high metabolic demands and dependence on electrical and chemical signals for transmitting and processing neural information. Therefore, the cerebral vasculature has a unique structural function as the blood–brain barrier (BBB). The structural basis of the BBB is microvascular endothelial cells, which, with astrocytes, basement membranes, pericytes, and neurons that are physically close to the endothelium, form the neurovascular unit (NVU). The corresponding cells, the accompanying junction complexes, and transport proteins constitute three main functions of the BBB: physical, transport, and metabolic barriers. These barriers strictly control the entry of water molecules, ions, proteins, lipids, and cells from the blood into brain tissues and promptly discharge and degrade metabolites or harmful substances in brain tissues to maintain brain microenvironment homeostasis and normal neurological functions. Ischemic stroke structurally and functionally disrupts the barrier function of the NVU, leading to BBB leakage and triggering a range of clinical symptoms. This review first deals with the interactions of BBB-related cell types/structures (endothelial cells, glial cells, pericytes, neurons, and extracellular matrix) in the NVU. This summary outlines the changes that occur during BBB disruption in ischemic stroke and the main regulatory mechanisms. Understanding normal BBB function and post-infarction changes in the BBB will help evaluate and treat ischemic stroke in the future.

## 2. Structural components of the BBB: neurovascular unit (NVU)

**Figure 1:**
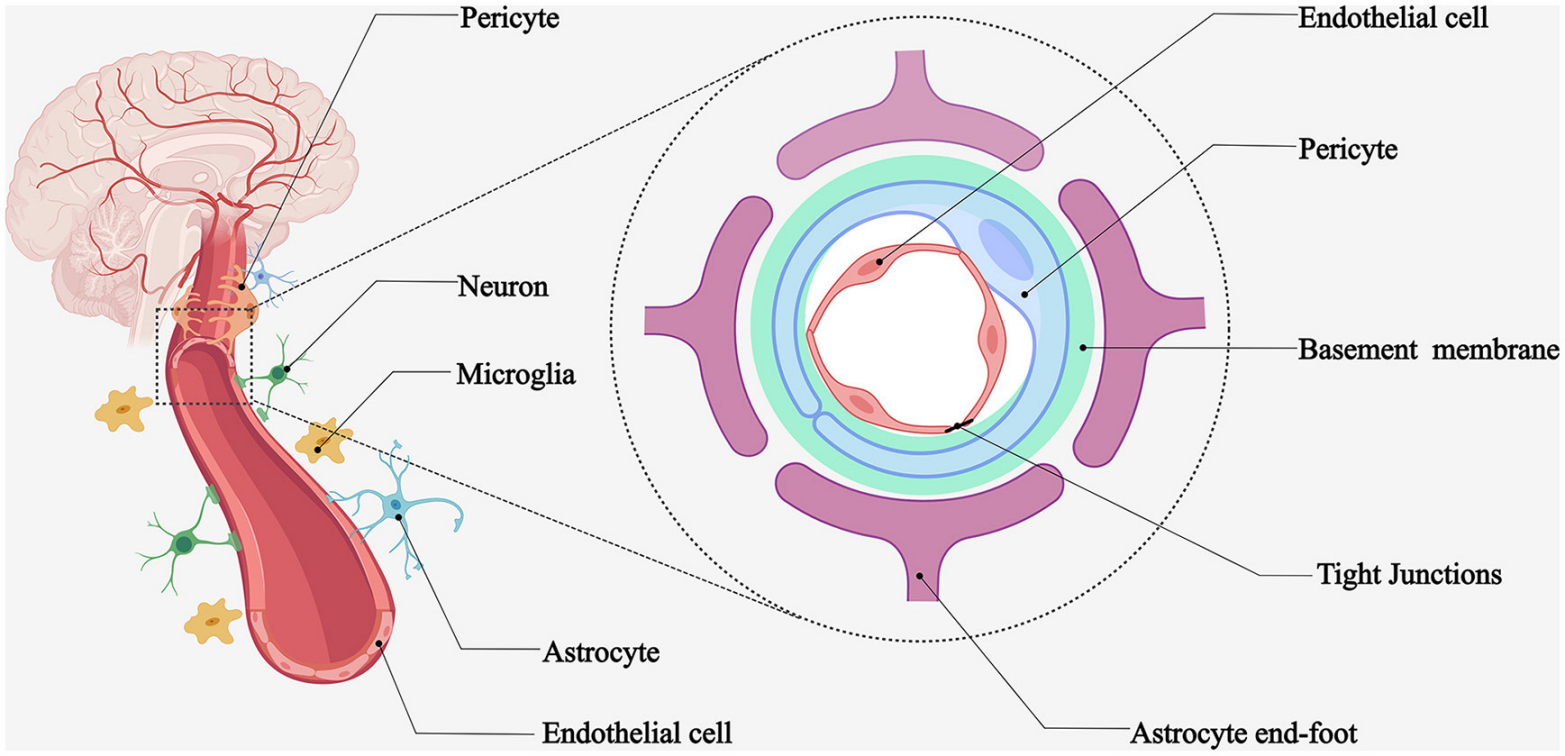
Schematic diagram of NVU. Microvessels adjacent to highly vascularized brain tissues provide blood flow to the surrounding nerve cells. Brain microvascular endothelial cells, pericytes, astrocytes, basement membrane, neurons, and microglia form the neurovascular unit.

NVU is a functional unit consisting of neurons and microvascular endothelial cells responsible for their blood supply, interacting with surrounding astrocytes and pericytes and regulating local blood flow (1). The cerebral vasculature has a very complex and delicate microscopic structure. First, brain tissues are highly vascularized, with at least one capillary supplying blood within 15 μm of each nerve cell (2), which can provide the nutrients required for neural activity and promptly eliminate metabolic waste. Second, cerebral small vessels and microvessels, surrounding vascular wall cells (smooth muscle cells and pericytes), astrocytes, basement membrane, and nerve cells, form the NVU microstructure (Figure 1). Some experts considered oligodendrocyte precursor cells and microglia as part of the NVU. The concept of the NVU was formally introduced at the 2001 Stroke Progress Review Group meeting of the National Institute of Neurological Disorders and Stroke (3), where neuroscientists emphasize the close relationship between nerve cells and blood vessels. The microstructure of the NVU is the cellular and molecular basis for many cerebrovascular-specific functions, such as neurovascular coupling and the BBB.

The NVU components work in concert to regulate microvascular permeability, ion homeostasis, nutrient transport, metabolic toxin efflux, and cerebral hemodynamics. Disruption of these components can lead to BBB dysfunction (4). Experimental evidence has demonstrated that the interaction between developing vascular and neural tissues is crucial for BBB development, interoperability, and symbiosis (5). Astrocyte end-feet, pericytes, microglia, and neuronal protrusions surround the brain microvasculature. Such close intercellular connections mediate the BBB-specific phenotype (1). Next, we provide an overview of these NVU components and highlight the features of ischemic stroke disruption.

### 2.1. Brain microvascular endothelial cells

The brain microvascular endothelial cells located at the junction of brain tissues and blood have essential physiological functions, including barrier function, nutrient transport, receptor-mediated signal transduction, and leukocyte infiltration (6). Endothelial cells are characterized by a lack of fenestrae and the expression of tight junction (TJ)–protein complexes between adjacent endothelial cells (7). In addition, Peterson et al. found that endothelial cells have mechanoreceptor properties that respond to absolute fluid and transmural pressure, contributing to cerebral blood flow autoregulation (8). As the first barrier for blood components entering the nervous system, endothelial cells are the core components of the BBB and have unique structural and molecular properties, including the following five main aspects (9): high expression of TJ proteins that control the paracellular pathway between endothelial cells: TJ proteins: occludin, claudin, zonula occludens (ZO)-1, ZO-2, ZO-3, cingulin, afadin-6 (AF-6), and 7H6; adherens junctions (AJs): cadherins and associated proteins directly linked to actin filaments (6) and junctional adhesion molecules (JAMs). Lower rates of transcytosis prevent blood components from crossing the BBB via non-specific vesicular transport. In stroke, endothelial vesicles increase, representing an increased transcellular response (10, 11). The high endothelial glycocalyx layer of the cell membrane reduces the passive diffusion of blood macromolecules across the BBB. Low expression of leukocyte adhesion molecules inhibits the adhesion and transit of blood immune cells into the brain. A comprehensive molecular transport system includes the high expression of several transporter proteins (transporting glucose, amino acids, ions, and lipid molecules), membrane receptors, and efflux proteins (multidrug resistance proteins, breast cancer resistance proteins, and multidrug resistance-associated proteins) for transporting material molecules between the blood and brain tissues (12, 13).

Owing to these properties, the BBB strictly limits the non-specific entry of blood components into the brain and efficiently provides the required nutrients to the neural tissue. The TJs between adjacent endothelial cells, specific transport systems on the luminal and abluminal endothelial cell membranes, and the distribution of metabolic enzymes constitute three barrier functions of the BBB: the physical, transport, and metabolic barriers, respectively. Physical barrier: the presence of TJs allows molecules to cross the BBB via a transcellular rather than a paracellular pathway (1, 7). AJ proteins, inhibition of non-selective window pores, cellular drinking, high flow across cells, and inhibition of leukocyte adhesion molecules are also involved in the physical barrier composition (14). Most molecules cross the BBB via the transcytosis pathway (15). In contrast, the paracellular pathway, which depends on the permeability gradient (16–21), accounts for only a small percentage of TJ limitations (18, 20, 22). Transport barrier: much lower rates of endocytosis/transcytosis, mainly through the transcellular pathway. Transcellular diffusion: diffusion locations are on the luminal and abluminal membranes of endothelial cells. They are constrained to gases such as O_2_ and CO_2_ and small lipophilic molecules < 400 Da (17, 19, 20, 23, 24). Carrier-mediated transport: active or passive substrate-specific transport (21, 24). Glucose transporter 1 (GLUT1), monocarboxylate transporter 1 (MCT1), L-type amino acid transporter 1 (LAT1), and major facilitator superfamily domain-containing protein 2a (Mfsd2a) carry glucose, lactic, amino, and fatty acids, respectively (25–29). GLUT1, a large neutral amino acid and nucleoside transporter protein, is essential for maintaining BBB homeostasis (30). Transcytosis remains the preferred pathway for the selective transport of plasma macromolecules such as albumin and low-density lipoproteins, despite limited transcytosis in CNS endothelial cells (31). Receptor-mediated transcytosis: major transport pathways for molecules without a specific carrier (30). These molecules bind to cell-surface receptors and form endocytic vesicles that cross the BBB (22). Adsorptive transcytosis: charge interaction between the compound and the luminal membrane of endothelial cells, inducing endocytosis (32). Efflux transport is concentrated on the luminal side of the membrane (29). To prevent the accumulation of toxic compounds across the BBB, substances are extracted from the nervous system into the bloodstream by moving a series of small molecules up to a concentration gradient (20, 21). For example, sodium-dependent amino acid transporters present in the endothelial cell membrane can remove amino acids from the brain, and these accumulated amino acids may evoke neurotoxicity during stroke (33–35). Brain microvascular endothelial cells are more abundant in the mitochondria than in the endothelial cells of other organs, which laterally reflect the energy demand that depends on adenosine triphosphate (ATP) transport (6, 36). Metabolic barrier: enzymes that constitute this barrier include ectoenzymes (peptidase and nucleotidases) and intracellular enzymes (monoamine oxidase and cytochrome p450) (37).

In contrast to the importance of TJ proteins, the role of cytosolic transport in regulating BBB permeability has been traditionally ignored or underestimated. Researchers have recognized the importance of cytosolic transport in the last few years, which has become a research hotspot in the BBB field. Low levels of cytosolic transport inhibit BBB permeability during embryonic development and adult physiological states, whereas elevated levels contribute significantly to BBB disruption under various pathological conditions, including chronic hypoperfusion, stroke, and cortical depolarization (38, 39).

Excellent and pioneering work in this area was carried out by Prof. Chenghua Gu's team at Harvard Medical School, who identified that Mfsd2a on the cell membrane is highly expressed in cerebrovascular endothelial cells, which promotes BBB formation and function mainly by maintaining a low rate of transcytosis and effectively inhibiting fossa-mediated cytosolic transport (40). Therefore, it reduces the non-specific transport of blood components into the brain and maintains the functional integrity of the BBB (41). Further studies have shown that the phospholipid transport function of Mfsd2a alters the lipid composition of endothelial cell membranes, thereby inhibiting cell membrane fossa formation, which inhibits fossa-mediated cytosolic transport (42). In addition, related studies have reported that Mfsd2a transports omega-3 fatty acids across CNS endothelial cells (43, 44). Future studies need to determine the relationship between these two functions (45, 46).

### 2.2. Astrocytes

Astrocytes coat more than 99% of the BBB endothelium (1, 5, 47, 48). Astrocyte end-feet form the BBB and maintain its integrity (1, 49, 50). They have multiple functions, including nutrition, structural support, BBB formation, neuronal metabolism, extracellular environment maintenance, cerebral blood flow regulation, intercellular communication stabilization, immune response control, neurotransmitter synthesis, and resistance to oxidative stress (51–54).

Astrocyte–endothelium interactions are essential for electrolyte homeostasis in the brain under both normal and pathological conditions (4). Perivascular astrocyte end-feet are highly structured and contain orthogonal arrays of particles, composed of the abundant water channel aquaporin-4 (AQP4) and the Kir4.1K^+^ channel, which are involved in ion volume regulation (1, 48, 55). AQP4 is associated with cytotoxic edema during ischemic stroke (4).

Astrocyte–endothelial interactions strengthen the TJ, minimize the gap junctional area of endothelial cells, and contribute to regulating cerebral blood flow (52). Both are involved in the induction and modulation of unique endothelial cell phenotypes (6, 56, 57). Astrocytes do not appear in the NVU until birth (14, 58). They are not directly involved in the physical properties of the BBB and may modulate its phenotype (6, 59). An experiment was conducted in 1969 to demonstrate this phenomenon. Horseradish peroxidase injected into the brain passes directly through astrocyte end-feet, and diffusion is blocked on the abluminal membrane of the endothelium. Studies over the past two decades have shown that astrocytes mediate barrier function, mainly by modifying the morphological composition and chemical expression of TJ (60, 61). The main molecules involved were TJ-related proteins, platelet endothelial cell adhesion molecule-1, ZO-1, and claudin-5 (60, 62). Astrocytes release several pro-barrier phenotype regulators (63), including nitric oxide (NO), which regulates vasodilation (64); sonic hedgehog, which governs TJ formation; and vascular endothelial growth factor (VEGF), which is associated with vasogenic edema in ischemic stroke (65). Astrocytes have different morphologies and phenotypes, depending on the region where they are located (66); 8 of the 11 common phenotypes were associated with vascular cross-talk (1). Multiple astrocyte morphologies can upregulate and strengthen these three barriers, making the TJ more robust, increasing the expression and polarization of transporter proteins, including Pgp24, and refining the enzyme system (67–69).

Similar to amino acid efflux transporters in endothelial cells, the excitatory amino acid transport proteins are expressed in astrocytes: excitatory amino acid transporters 1 and 2. These proteins remove extra glutamate from synapses and maintain the relative stability of excitatory neurotransmitters in the brain (70). Astrocytes play an important role in ischemic stroke-induced neuroinflammation.

### 2.3. Pericytes

Pericytes are kidney-shaped cells with nuclei that protrude from the lumen. They contain a small cytoplasm and are distributed irregularly along the brain microvessel wall (71–74). Pericytes cover at least 80% of the brain microvascular wall, and cell protrusions emanating from pericytes penetrate the cellular matrix to cover 20–30% of the microvascular perimeter (6). Such dimensional contact with the vasculature serves as a support for the endothelium of the NVU. It plays important physiological roles in endothelial cell development (6), BBB formation and integrity maintenance, vascular maturation and stabilization, and immune cell trafficking.

Experimental evidence suggests that pericytes have contractile properties and that contractile pericytes–coat cerebral microvessels (71, 72, 75) regulate capillary blood flow and influence cerebral blood flow autoregulation. A recent scRNA-seq study identified the molecular mechanisms by which pericytes regulate vessel diameter. Strong evidence for the role of pericytes in controlling vessel diameter is provided by the presence of receptors for vasoactive factors, such as L-type voltage-gated calcium channels, and those involved in the contraction of smooth muscle cell actin on pericytes (76). To a certain extent, pericytes act as the “vascular smooth muscle cells” of the brain's microvasculature.

Evidence for pericyte–endothelial cell interactions has also been found. The absence of pericytes in mice by experimental means revealed the downregulation of Mfsd2a expression (43). Pericyte deletion leads to increased endothelial transcytosis without affecting the integrity of TJs (59, 77). Recent studies have shown that pericytes regulate endothelial transcytosis by binding to integrins in endothelial cells through vitronectin expression (78).

### 2.4. Neurons

As mentioned previously, neurons and their nearby cerebral microvasculature are in close contact, and there is an intimate interaction between neurons and their matching vessels. Although direct neuron–endothelial contact has been demonstrated (66, 79), neuronal communication in the NVU is primarily mediated by astrocytes (80). Little is known about the other structural and functional contributions of neurons to the BBB (80). The suggested pathological factors, such as ischemic stroke-induced BBB disruption, are anatomical and usually accompanied by selective compensatory changes in cerebral blood flow (81–83). This indicates that interactions between neurons and microvessels can modulate BBB permeability.

### 2.5. Extracellular matrix (ECM)

The ECM layer generated by the basal cell membrane serves as a signaling site for cell-cell interactions (1). The ECM is divided into two layers: the vascular basement membrane produced by pericytes and endothelial cells and the glial basement membrane secreted by astrocytes (84). The structural proteins that comprise the ECM include collagen type IV, laminin, fibronectin, elastin, thrombospondin, and various proteoglycans that are readily degraded by proteases. Their degradation is associated with increased BBB permeability during ischemic stroke (85, 86). The ECM wraps around endothelial cells and pericytes. It separates and anchors cells through adhesion receptors. Astrocytes and pericytes secrete integrin and dystroglycan families of matrix adhesion receptors that are distributed in the ECM and mediate NVU function (87).

### 2.6. Tight junction (TJ)

TJ is an essential junction complex between adjacent epithelial cells. TJs comprise intact membrane and cytoplasmic accessory proteins, including occludin, claudins, JAMs, ZO proteins, and cingulin. Actin is a cytoskeletal protein that maintains endothelial structure and function. TJ proteins are linked to cytoskeletal actin by accessory proteins. The tightness of the TJ connections determines the paracellular permeability of water-soluble molecules (6, 49). The BBB transendothelial electrical resistance (TEER) is a measure of the degree of permeability and efficiency of TJs in the BBB (88). The TEER of peripheral microvessels is typically 2–20 ohm.cm^2^, while the TEER of the brain endothelium can reach 1,000 ohm.cm^2^ (1). The polarity of the luminal and abluminal membranes of endothelial cells contributes to barrier function. The concept of polarity is derived from quantitative biochemical studies (6). TJs perform a structural gate function (1), limiting paracellular permeability. Its fence function separates the apical and basal regions of the cell membrane (89), allowing endothelial cells to exhibit apical–basal polarization properties. The luminal and abluminal polarization of receptors, ion channels, and enzymes in endothelial cells makes the functional partitioning of endothelial cells more refined and rational (6). TJ proteins exist in different isoforms because of their tissue origins (4). Phosphorylation regulates the activities of TJ and accessory proteins (5).

### 2.7. Occludin

Occludin is a 65-KDa protein with 504 amino acids (6, 90). Occludin has two extracellular loops and one intracellular loop, and its carboxyl and amino terminals are anchored to the cytoplasm. The carboxy-terminal serine residues of occludin are linked to the cytoskeleton via ZO-1 and ZO-2, allowing occludin to bind to the basal cellular membrane. Occludin phosphorylation regulates its functional state (91). The volume of occludin in endothelial cells is much higher in the CNS than in the periphery (90, 92). Recent studies have shown that occludin is a key factor in regulating BBB permeability (93–96). A study in 2017 using a rat model of ischemic stroke found that the occludin protein in TJs of brain microvessels caused by cerebral ischemia rapidly degraded. Elevated blood occludin levels show a time-dependent correlation with the degree of BBB injury. Therefore, occludin proteins in the blood may serve as a clinically relevant marker of ischemic stroke (97).

### 2.8. Claudins

Claudins comprise numerous proteins. They have two extracellular loops through which adjacent endothelial cells are connected, forming the major sealing component of TJs (98). The two intracellular loops of claudins can bind to ZO-1, ZO-2, and ZO-3 through their carboxyl termini (99, 100). Claudins are morphologically similar to occludins but do not share sequence homology (90). Claudin-5 plays a crucial role in the early development of the CNS as a symbol of the BBB (101). The phosphorylation pathway can regulate the functional activity of claudin-5 (102, 103). The phosphorylation of claudin-5 at Thr207 increases the permeability of TJ. In experimental models of pathological conditions, such as ischemic stroke, claudin-1 is lost in the cerebral vasculature (49), claudin-5 expression is reduced (104, 105), and the interaction between claudin-5 and occludins is disrupted (106).

### 2.9. Junctional adhesion molecule (JAM)

JAMs are a family of immunoglobulin superfamily proteins that localize to intercellular slits. JAM-A, JAM-B, and JAM-C are expressed in endothelial cells and are involved in the construction and maintenance of the TJ (4, 107). The extracellular portion of the transmembrane structural domain of JAMs consists of two immunoglobulin-like loops formed by disulfide bonds (107). JAMs mainly bind to the intracellular components ZO-1, partitioning defective protein-3(PAR-3), AF-6, and multi-PDZ-protein-1(MUPP-1) (108). Like claudins, JAMs participate in homophilic and heterophilic interactions between cells (107). Deficiency in JAM protein expression and BBB disruption are directly related (109–112).

### 2.10. Membrane-associated guanylate kinase (MAGUK) proteins

MAGUK proteins are also called cytoplasmic accessory proteins. MAGUK proteins are accessory components of TJ structures. Their multiple structural domains are involved in protein–protein interactions (113), forming protein complexes attached to the cell membrane. The MAGUK proteins associated with TJ are ZO-1, ZO-2, ZO-3, and the newly identified cingulin, 7H6, and AF-6 (5, 113). ZO-1, a 220-KDa phosphoprotein, is expressed in epithelial and endothelial cells (114, 115). ZO-1 bridges the TJ proteins with the actin cytoskeleton (91, 116–118). ZO-1 transmits the TJ state to the intracellular zone and vice versa. Decreased ZO-1 expression is associated with increased BBB permeability (118). ZO-2, a 160-KDa phosphoprotein, shares homology with ZO-1 (119, 120). Immunofluorescence microscopy studies conducted in 2003 showed that ZO-3 was enriched in the TJ of epithelial cells (121). Although it is expressed in the epithelium, its role in TJ has not been fully elucidated (122). Cingulin is a 140–160-KDa phosphoprotein. It is localized to the cytoplasmic surface of TJs and is associated with the ZO proteins, myosin, JAM-A, and AF-6 (123, 124). As a vital scaffolding protein of TJs, it can form various junctions and transmit mechanical forces generated by cytoskeletal contraction, thus regulating BBB permeability (5, 125). 7H6, a 155-KDa phosphoprotein, reversibly detaches from TJ when ATP is depleted, increasing paracellular permeability (126, 127). AF-6, a 180-KDa protein, is involved in TJ regulation by linking it to ZO-1 (103).

### 2.11. Adherens junction (AJ)

AJ: An intercellular junction, also called zonula adherens (1), is a specific type of intercellular interaction consisting of cadherins, catenins, vinculin, and actinin (128). However, the effect of AJ on BBB permeability in pathological states has not been fully elucidated. However, its interaction with vascular endothelial growth factor (VEGF) receptor-2 and vascular endothelial (VE)-cadherin-mediated upregulation of claudin-5 suggests that AJ plays a central role in angiogenesis and the regulation of TJ integrity (129, 130).

## 3. Ischemic stroke

Ischemic stroke is a leading cause of death and disability worldwide. It imposes substantial economic and emotional burdens on patients, families, and society. Ischemic stroke is characterized by a sudden reduction or cessation of oxygen and blood supply due to local arterial occlusion of the brain tissues, resulting in irreversible cell death and tissue damage in the infarct core. The surrounding penumbra may regain function by restoring cerebral blood flow owing to mild ischemia. Various functions of the cerebral vasculature, such as BBB and blood flow regulation, are also affected by acute ischemic–hypoxic injury. Disruption of the BBB is a hallmark of ischemic stroke (131). The mechanisms of BBB disruption induced by stroke include phosphorylation of TJ proteins, regulation of transporter protein expression, neuroinflammation, and abnormal enzyme function (132).

### 3.1. Ischemic phase and biphasic nature of barrier permeability

Ischemic stroke occurs chronologically during the ischemic and reperfusion phases, during which a series of cellular changes occur (4). The BBB is incomplete long before neuronal damage occurs (133, 134), and the concept of NVU emphasizes that BBB disruption in ischemic stroke damages the cell structure and disrupts intercellular interactions (135, 136). Although called a barrier, the BBB is not a physical wall (137). In fact, the breakdown of the BBB caused by brain infarction is a dysregulation of molecular proteins associated with its three functional barrier properties.

Blockage of cerebral blood flow and rapid hypoxia in the ischemic core triggers a chain of events in the infarct area, including ATP depletion; neurotransmitter leakage: dopamine, excitotoxic glutamate, arachidonic acid, and ceramide efflux that produce extremely acute neurotoxic effects; ion imbalance: increased intracellular calcium; metabolic disorders and increased acidosis; endothelial cells, astrocytes, neurons swelling due to lactic acid accumulation; cerebral microvascular diameter reduction; oxidative stress; neuroinflammation activation; protease induction and promotion of extracellular matrix degradation at the BBB (138, 139). Hypoxia disrupts the localization or expression of TJ proteins in several *in vitro* models of cerebral infarction (118, 140). Fischer et al. demonstrated that ZO-1 and ZO-2 translocate to the nucleus in a hypoxic environment *in vitro* (141). In addition, the *in vitro* hypoxic environment altered the cell membrane localization and expression of claudin-5, whereas TEER decreased (142). To date, there have been some new developments regarding BBB disruption after ischemic stroke. In 2014, Knowland et al. observed that barrier function was impaired as early as 6 h after stroke, with increased endothelial vesicles and transcytosis in a transgenic mouse stroke model. In contrast, the TJ showed severe structural defects after only 2 days. This suggests that early BBB defects are caused by defective transport barriers (10). Data from a study by Haley and Lawrence subsequently demonstrated that endothelial vesicle upregulation might exacerbate BBB permeability in the early stages of cerebral ischemia and that the number of endothelial vesicles correlates with the degree of BBB disruption (143). Krueger et al. established various experimental models of focal cerebral ischemia, and their experimental data strongly suggest that ischemia-associated BBB deficits are primarily caused by endothelial cell degeneration (144).

**Figure 2:**
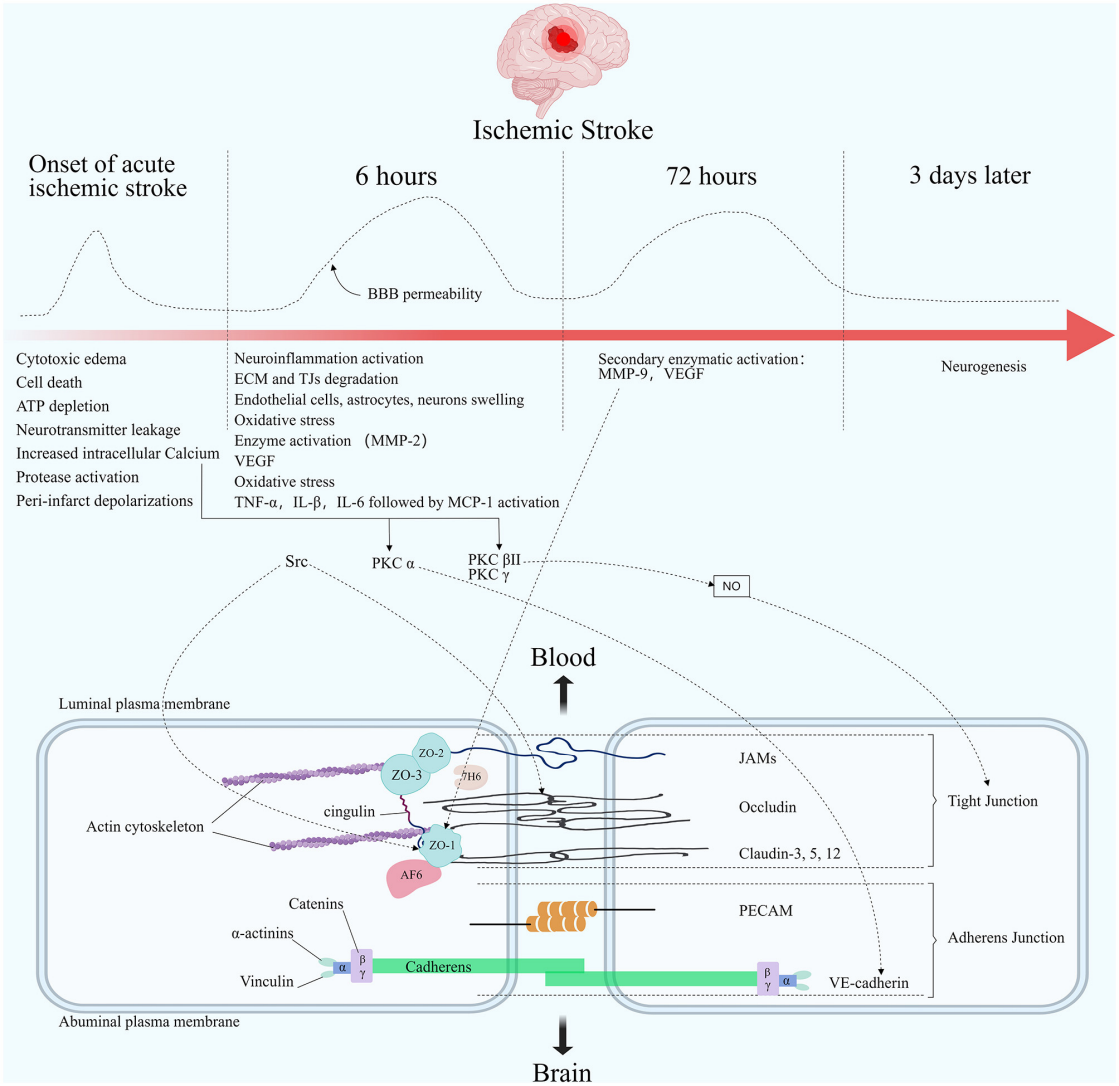
Molecular composition of BBB tight junctions and major changes in acute ischemic stroke. The TJ consists of three integral membrane proteins, claudins, occludin, and junction adhesion molecules (JAMs), and a number of cytoplasmic accessory proteins including zonula occludens 1, 2, and 3 (ZO-1, 2, 3), cingulin, afadin-6 (AF-6), and 7H6. Membrane proteins are connected to actin by cytoplasmic proteins. The carboxy-terminal serine residues of occludin are linked to the cytoskeleton via ZO-1 and ZO-2. Adherens junctions are formed by cadherins, catenins, vinculin, and actinin. Among the important molecules are vascular endothelial cadherin (VE-cadherin) and platelet endothelial cell adhesion molecules (PECAM). The figure shows the changes in blood-brain barrier permeability at different times after the onset of ischemic stroke, as well as the main pathophysiological processes in each stage. Acutely elevated local cerebral blood flow can lead to initial reperfusion permeability. The opening of the BBB presents a biphasic phenomenon. The first phase usually occurs within 6 h after an acute cerebral infarction. The second phase usually occurs within 72 h after the onset of an acute cerebral infarction. Elevated Ca^2+^ concentration in the endothelial under ischemic hypoxic conditions regulates TJ proteins' stability through multiple pathways. PKC α can act on VE-cadherin to participate in AJ degradation. PKC βII and PKC γ cause TJ changes by regulating NO activity. Src phosphorylates occludin and ZO-1. Elevated MMP-9 reduces ZO-1 expression and its cytoplasmic translocation, leading to barrier disruption.

Opening of the BBB is a biphasic phenomenon in preclinical models of ischemic stroke (145). First, after the onset of acute ischemic stroke, initial reperfusion permeability can occur at the time of acute elevation of the regional cerebral blood flow (rCBF), followed by biphasic permeability of the BBB (4). Animal models and human studies have documented that the first phase occurs in the hyperacute phase of acute ischemic stroke, usually within 6 h of onset (146–149). The second phase occurs in the acute phase of acute ischemic stroke, usually within 72/96 h after onset, when the initial cytotoxicity-induced neuroinflammation further disrupts the BBB. A second peak infiltration rate is observed during this phase (146–148, 150). It is worth noting that other studies, including those in humans, have shown that elevated BBB permeability can persist for several weeks after stroke (149, 151) (Figure 2).

### 3.2. Brain edema

Ischemic brain injury can rapidly lead to cerebral edema, with excessive fluid retention in the intracellular or extracellular spaces of the brain (36). Cerebral edema is a leading cause of clinical deterioration and death in ischemic stroke and is divided into cytotoxic and vasogenic edema (152, 153). Cytotoxic edema appears within minutes of ischemia, after which the BBB breaks down and vasogenic edema emerges (154, 155). The dysfunction of ion transporters in the BBB is an important mechanism that leads to brain edema. Cytotoxic edema: after ischemia, the activity of Na^+^/H^+^ exchangers, Na^+^-K^+^-Cl cotransporters, and calcium-activated potassium channel KCa3.1 at the BBB increases, facilitating the transport of Na^+^ and Cl^−^ across the cells into the relatively intact BBB (156–158). Cerebral edema gradually develops as Na^+^ intake increases (156, 157). The endothelial cells showed initial nuclear swelling and moderate cytoplasmic swelling within 2 h. Swelling of astrocytic end-feet begins within 5 min of energy depletion after cerebral infarction, leading to the detachment of the end-feet from the endothelium (159–161). Tagaya observed that 2 h after middle cerebral artery occlusion, there was no expression of α1β1, α3β1, and α6β1 on endothelial cells and α6β4 on astrocytes (162). Vasogenic edema arises from changes in the TJ, which increase the permeability of macromolecules and cause fluid to flow outside the blood vessels (163).

AQP, a highly permeable water channel, is highly enriched in the astrocyte end-feet. It is involved in maintaining fluid homeostasis in the brain. In several ischemic stroke models, AQP4 contributes to brain edema formation, and its absence disables cellular water uptake (164, 165). The structure of the NVU determines the complexity of BBB disruption induced by ischemic stroke, and there is no general way to regulate it. The main modulators of this process are described below. Studies on TJ protein modifications after ischemic stroke have focused on phosphorylation (36). Phosphorylation is the primary regulatory mechanism of protein effects on the BBB (5). The phosphorylation and dephosphorylation states of TJs depend on the amino acid residues that undergo phosphorylation and the type of stimulus (6). Some functionally relevant protein kinases are summarized below.

### 3.3. Related protein kinases

Calcium-dependent protein kinase C (PKC) subtypes may be involved in TJ regulation, including α, βI, βII, and γ. Myosin light-chain (MLC) phosphorylation regulates endothelial cell paracellular permeability. MLC phosphorylation leads to actin–myosin contraction via calcium/calmodulin-independent MLC-kinase (MLCK) phosphorylation (166, 167). This process increases the permeability of the TJ, allowing F-actin, ZO-1, and occludins to redistribute (168). PKC inhibitors can reduce endothelial cell permeability by introducing several factors (thrombin, bradykinin, VEGF, hydrogen peroxide, platelet-activating factor, and neutrophils) (169, 170). The expression of PKC α, βI, and ε shows an increase in hypoxic or hyperglycemic environments (171). Following ischemia, hyperglycemia, and inflammatory insults, endothelial cells exhibit increased permeability via a PKC α-dependent pathway (172–175). PKC α is also involved in the catabolism of AJs via VE-cadherin, which leads to increased paracellular permeability (175). Increased expression of PKC βII and PKC γ can regulate NO synthase, indicating that PKC may affect TJ changes by regulating NO activity (176). Mitogen-activated protein kinases (MAPKs) belong to the serine/threonine kinases family. The most studied MAPKs are extracellular signal-related kinases (ERK1/2), c-Jun N-terminal kinases (JNK), and p38-protein (170). Studies in consecutive years have demonstrated that the integrity of TJ is associated with increased levels of cyclic adenosine monophosphate (cAMP) (177–179). The effect of cAMP is most likely mediated by the protein kinase A (PKA) pathway. PKA stabilizes cytoskeletal proteins, mediates the dephosphorylation of MLC, enhances cell-matrix adhesion, and detaches F-actin from myosin (180–182). Protein kinase G (PKG) is a serine/threonine-specific protein kinase activated by cyclic guanosine monophosphate (cGMP). Both *in vivo* and *in vitro* studies have demonstrated that factors relevant to ischemic stroke, including bradykinin, histamine, NO, tumor necrosis factor (TNF)-α, platelet-activating factor, and VEGF, can lead to increased NO-cGMP dependent paracellular permeability (141, 170, 183). Protein tyrosine kinases (PTKs) act as intracellular signal transduction molecules that regulate endothelial paracellular permeability. PTK can be classified as receptor and non-receptor-mediated types. Occludin phosphorylation diminishes its ability to bind to ZO-1 and ZO-2, leading to increased paracellular permeability (140, 184, 185). Phosphorylation of AJ proteins unanchors them to the cytoskeleton and affects TJ permeability (186). Among the non-receptor-mediated PTKs, the Src family plays a vital role in TJ regulation (187, 188). Interleukin (IL)-1β, reactive oxygen species (ROS), and tumor necrosis factor (TNF) modulate Src and increase endothelial permeability of the BBB (189–191). Phosphorylation of myosin MLCK by Src enhances actin-myosin interactions and increases paracellular permeability (192, 193). Studies have demonstrated that cerebral infarction-related oxidative stress-induced endothelial cell permeability is associated with Src, which phosphorylates occludin and ZO-1 (184, 194, 195). Oxidative stress can also lead to F-actin redistribution and stress fiber formation (196, 197).

### 3.4. Ca^2+^

Under physiological and pathological conditions, TJ responds rapidly to intracellular signals that regulate junctional complexes (6). Molecules that regulate BBB permeability are induced by altering intracellular Ca^2+^ (49). Early studies have demonstrated that Ca^2+^, a second messenger, is a key component in the regulation of TJ through multiple pathways (such as PKC, MAPK, and phospholipase-A2) (198). Elevated intracellular Ca^2+^ triggers a signaling cascade response that regulates TJ transcriptional expression and alters TJ post-translational distribution (199). The endothelial Ca^2+^ concentration increases in an ischemic–hypoxic environment, the ATP-dependent efflux mechanism is disrupted, and the control of intracellular Ca^2+^ by the cytoplasmic endoplasmic reticulum Ca^2+^-ATPase fails (199, 200). Increased Ca^2+^ in endothelial cells enables the activation of MLCK (166, 167), induces actin reorganization, changes cell shape, and increases cell permeability (201, 202). Ca^2+^ directly affects the stability of BBB endothelial cell membranes and TJ proteins by promoting phospholipase-A2 activation, which allows membrane phospholipids to release free fatty acids (4).

### 3.5. Neuroinflammation

Neuroinflammation is an integral part of the pathophysiology of cerebrovascular diseases, particularly ischemic stroke. Many studies have shown that poststroke neuroinflammation is an important factor in the long-term prognosis of ischemic stroke. After a stroke, various factors, such as ROS formation, necrotic cells, and damaged tissues, can activate inflammatory cells, resulting in an inflammatory response. Several substances have been found in the cerebrospinal fluid (CSF) of stroke patients, and studies suggest that TNF-α and IL-1β initiate neuroinflammation in ischemic stroke (4). Cyclooxygenase inhibition can eliminate TNF-α-, IL-1β-, and IL-6-induced increase in endothelial paracellular permeability, suggesting a role for arachidonic acid in increasing BBB permeability during ischemic stroke (203, 204). The production and release of chemokines chemoattractant protein monocyte chemoattractant protein-1 (MCP-1) and cytokine-induced neutrophil chemoattractant (CINC) are regulated by regulatory factors. In the pathological context of stroke, MCP-1 is a major factor associated with leukocyte entry into the brain (205). The biphasic BBB permeability that occurs during hypoxic reperfusion is consistent with an increase in the secretion of MCP-1 by astrocytes and endothelial cells in the brain (206). Biphasic BBB permeability is associated with the redistribution of occludin, ZO-1, ZO-2, and claudin-5 (206). IL-1 is associated with the induction of endothelial cell adhesion molecules during ischemic stroke. IL-β-induced increase in paracellular permeability of the BBB is related to the loss of occludin and ZO-1 in the junctional complex (207).

### 3.6. Enzyme activities

Matrix metalloproteinases (MMPs) are a family of zinc-dependent endopeptidases that can degrade fibronectin and laminin (50) and have been identified as key factors in BBB disruption in ischemic stroke (208). The basement membrane is a scaffold for brain endothelial cells and is composed of type IV collagen, laminin, fibronectin, elastin, thrombospondins, various proteoglycans, and heparin sulfate. MMPs exhibit substrate specificity for type IV collagen, laminin, and fibronectin, which degrade basement membranes. MMPs have been identified as the apparent initiators of BBB disruption. After the onset of ischemic stroke, MMP expression is upregulated and activated through pro-inflammatory cytokine pathways (via NF-kB) or activation of HIF-1a and furin (50, 209). A clinical study showed that MMP-9 levels were significantly elevated in patients with acute ischemic stroke (210). MMP-mediated BBB opening in ischemic stroke may be regulated by NO signaling. The activation of MMP-2 is thought to initiate TJ protein degradation (211). In the late stages of stroke-induced BBB disruption, MMP-9 expression triggers devastating injury (211). The reason for the disruption of barrier function caused by elevated MMP-2/9 levels is the reduced expression of ZO-1 and its cytoplasmic translocation (212). Another study confirmed that the degree of BBB damage is consistent with reduced MMP-2/9 expression (213). Therefore, inhibition of MMPs may benefit patients with stroke (214, 215). Notably, despite their destructive effects in the acute phase of stroke, MMPs may play a beneficial role in the recovery phase of stroke (216–218). MMP-9 is upregulated in the peri-infarct region 7–14 days after stroke and may be involved in vascular remodeling. Interestingly another study demonstrated that MMP-13 promotes stroke injury in the acute phase but improves angiogenesis during the repair phase (217).

### 3.7. Actin polymerization

Actin is not a tight junction protein by traditional definition, but it has an important role in BBB stabilization (219). The actin cytoskeleton normally provides anchoring sites for tight junction proteins (220, 221). Dynamic stabilization between the cytoskeleton and tight junction proteins is important for BBB maintenance (222, 223). Actin is usually distributed uniformly throughout the endothelial cell in the form of short filaments and diffuse monomers (220). Unpolymerized globular actin polymerizes into the filamentous-actin (F-actin) form via an ATP-promoted process (224). Phosphorylation of myosin light chain (MLC) promotes the formation of dense stress fibers from short F-actins and induces actin contraction (225, 226). After ischemia and hypoxia, the normal actin cytoskeleton polymerizes into linear stress fibers spanning the endothelial cell interior (220). Increased contraction and tension of the actin cytoskeleton after polymerization occurs, leading to cell shrinkage, impaired tight junctions, and ultimately disruption of the BBB (225–227). Experimental evidence shows that actin polymerization after acute ischemic stroke induces the formation of stress fibers in the cytoskeleton, exposing the BBB to greater vulnerability to damage by MMPs (221). MMP-9 is usually considered to be the initiating factor of BBB disruption after acute ischemic stroke (228). Contrary to previous perceptions, the tension generated by stress fibers promotes the breakdown of tight junction proteins (229). Preliminary studies have shown that long before MMP-9 begins to degrade tight junctions, the redistribution of junctional complex proteins induced by actin polymerization causes structural changes in endothelial cells that disrupt the BBB (221). The structural disruption makes BBB more susceptible to degradation by MMP-9. Therefore, slowing down the cytoskeletal structural changes of endothelial cells in the early phase of ischemic stroke may bring a new therapeutic target for the protection of the BBB (230, 231).

### 3.8. Zinc

Zinc is the second most abundant essential trace element in the human body (232). It plays a crucial role in brain growth and development as a neurotransmitter or neuromodulator. Imbalances in zinc are closely associated with brain disorders (233). Most of the zinc ions in the human body are bound to zinc-binding proteins, while another portion exists as free zinc ions in organs, tissues, body fluids, and secretions (234). Zinc transport proteins regulate and maintain zinc ion homeostasis. Zinc-regulated transporter (ZRT)/iron-regulated transporter (IRT)-like protein (ZIP) family increases zinc intake, while the zinc transporter family (ZnTs) mediates the efflux of zinc from the cytoplasm, increasing the plasma zinc concentration.

BBB regulates zinc homeostasis in the brain. Studies have shown that under normal physiological conditions, the BBB isolates zinc between the plasma and the brain (235). Studies in animal models have shown that zinc homeostasis is critical for BBB integrity. Both zinc deficiency and zinc overload lead to BBB disruption under pathological conditions. In the experiment, dynamic magnetic resonance imaging measurements revealed that zinc deficiency leads to a significant increase in BBB permeability in rats exposed to high oxygen levels (236). In a rat model of cerebral ischemia, accumulation of zinc in ischemic microvessels was observed, leading to the loss of tight junction proteins (occludin and claudin-5). Accumulated zinc mediates cerebral ischemia-induced BBB injury by upregulating superoxide and MMPs. Chelated zinc reduces BBB permeability in ischemic rats (237). Interestingly, during the recovery phase of cerebral ischemia, zinc has been shown to alleviate brain ischemic atrophy, promote neural function restoration, and facilitate angiogenesis in the process of cerebral ischemic repair through the astrocyte-mediated HIF-1α/VEGF signaling pathway (238). A study compared the serum zinc concentration levels between ischemic stroke patients and healthy controls, and the results showed a significant decrease in serum zinc concentration in stroke patients. Zinc may represent an independent risk factor for stroke (239). Another study compared the serum zinc concentrations in patients with acute ischemic stroke to those with transient ischemic attacks. The results showed that stroke patients had significantly lower serum zinc concentrations (240). The fate of reduced serum zinc needs further study. MicroRNA-30a (miR-30a) is a member of the miR-30 family and is abundant in human endothelial cells. Experiments in cellular and animal models of ischemic stroke demonstrated that miR-30a can mediate BBB damage. miR-30a can directly negatively regulate ZnT4. Inhibition of miR-30a decreases BBB permeability, prevents degradation of tight junction proteins, and reduces intracellular free zinc in endothelial cells (241). This suggests that miR-30a may be an effective therapeutic target for ischemic stroke.

## 4. Discussion

Over the past 40 years, studies from animal models to clinical studies have elucidated the occurrence, development, composition, and maintenance of the BBB. The emergence of the NVU concept has dramatically changed the field of cerebrovascular research, prompting researchers to study and understand BBB physiology and pathology from a systematic perspective. We now recognize that the BBB is more than a simple physical anatomical barrier; endothelial cells, astrocytes, and other cells work together as functional complexes to regulate the barrier function. The basic structure of the BBB is now well understood; however, new insights into its fine structure are emerging. New technologies, such as two-photon microscopy, may be used in future studies for further in-depth investigation (41, 59, 77). Interactions between the components of the BBB are intricate. As mentioned above, many common points of phosphorylation are concentrated in the BBB cytoskeleton. We need to identify the processes that regulate the barrier and the core of the pathway. The recently discovered Mfsd2a protein is a current research hotspot, and further clarification of its role in physiology and pathology will help us better understand the BBB (41, 42). Future preclinical studies should consider multiple cell types in the NVU and the effects of peripheral systems on the NVU.

To date, the mechanism of BBB disruption in ischemic stroke remains unclear, and significant discoveries have been made regarding barrier permeability after disruption, such as the discovery of biphasic barrier permeability. However, the timing of the occurrence and maintenance of biphasic permeability must be clarified to provide a basis for the timing of stroke treatment. We also need an *in vitro* system to reliably reproduce the *in vivo* barrier, in which changes in the BBB can be dynamically observed using a stroke model. This ideal *in vitro* barrier requires the expression of essential components; however, the endothelial cells of the CNS can quickly lose their barrier properties in culture (242, 243). Recently, different research teams have made progress in perfecting the *in vitro* BBB model. In 2011, Hatherell et al. co-cultured endothelial cells with astrocytes and pericytes to recapitulate NVUs *in vitro*, increasing TEER (244). In 2013, Paolinelli et al. demonstrated that activation of the Wnt/β-catenin pathway *in vitro* increased the restrictiveness of endothelial cell monolayers without the need for co-culture with other cell types (245). Lippmann's study showed that endothelial cells with barrier properties can be generated from human pluripotent stem cells (246, 247). These new developments provide insights for the establishment of *in vitro* models. *In vitro* BBB models derived from stem cell sources may provide an ideal experimental setting for future studies.

In ischemic stroke-induced BBB disruption, the destructive factors may exhibit protective effects at different times, for example, MMPs. We need to clarify the role of these “dual nature” factors at different times to inform the timing of treatment for cerebral infarction. Studies on BBB disruption have revealed that endogenous barrier transport proteins may be potential targets for CNS drug transport (248). Neuronal protection alone may not improve the neurological prognosis for treating cerebral infarction. Therefore, therapeutic approaches that favor multiple cell types must be considered. Astrocytes are one of the most abundant and widely exposed cellular components. Thus, these compounds can potentially serve as therapeutic targets (52).

These detectable components are clinically valuable when structural proteins leak into the blood or CSF following BBB disruption after a stroke. For example, CSF albumin, IgG per serum albumin, or IgG ratio measurement can be used as clinical biomarkers to assess the integrity of the BBB (249–253). Serum S100B is a protein expressed by astrocytes in the brain and is considered a possible candidate for detecting BBB damage caused by astrocytes (254). Future research should focus on finding easily detectable BBB-related markers of cerebral infarction injury to provide a new basis for diagnosis and treatment.

## Author contributions

SX drafted the manuscript. SX, XZ, Z-HY, and X-KS illustrated the manuscript. XS revised the final manuscript. All authors have read and agreed to the published version of the manuscript.
